# Pterostilbene attenuates intrauterine growth retardation-induced colon inflammation in piglets by modulating endoplasmic reticulum stress and autophagy

**DOI:** 10.1186/s40104-022-00780-6

**Published:** 2022-11-04

**Authors:** Yanan Chen, Hao Zhang, Yue Li, Shuli Ji, Peilu Jia, Tian Wang

**Affiliations:** 1 College of Animal Science and Technology, Nanjing Agricultural University, Nanjing, 210095 China; 2 Institute of Animal Science, Jiangsu Academy of Agricultural Sciences, Nanjing, 210014 China

**Keywords:** Autophagic flux, Colon inflammation, Endoplasmic reticulum stress, Intrauterine growth retardation, Piglets

## Abstract

**Background:**

Endoplasmic reticulum (ER) stress and autophagy are implicated in the pathophysiology of intestinal inflammation; however, their roles in intrauterine growth retardation (IUGR)-induced colon inflammation are unclear. This study explored the protective effects of natural stilbene pterostilbene on colon inflammation using the IUGR piglets and the tumor necrosis factor alpha (TNF-α)-treated human colonic epithelial cells (Caco-2) by targeting ER stress and autophagy.

**Results:**

Both the IUGR colon and the TNF-α-treated Caco-2 cells exhibited inflammatory responses, ER stress, and impaired autophagic flux (*P* < 0.05). The ER stress inducer tunicamycin and the autophagy inhibitor 3-methyladenine further augmented inflammatory responses and apoptosis in the TNF-α-treated Caco-2 cells (*P* < 0.05). Conversely, pterostilbene inhibited ER stress and restored autophagic flux in the IUGR colon and the TNF-α-treated cells (*P* < 0.05). Pterostilbene also prevented the release of inflammatory cytokines and nuclear translocation of nuclear factor kappa B p65, reduced intestinal permeability and cell apoptosis, and facilitated the expression of intestinal tight junction proteins in the IUGR colon and the TNF-α-treated cells (*P* < 0.05). Importantly, treatment with tunicamycin or autophagosome-lysosome binding inhibitor chloroquine blocked the positive effects of pterostilbene on inflammatory response, cell apoptosis, and intestinal barrier function in the TNF-α-exposed Caco-2 cells (*P* < 0.05).

**Conclusion:**

Pterostilbene mitigates ER stress and promotes autophagic flux, thereby improving colon inflammation and barrier dysfunction in the IUGR piglets and the TNF-α-treated Caco-2 cells.

**Supplementary Information:**

The online version contains supplementary material available at 10.1186/s40104-022-00780-6.

## Introduction

Intrauterine growth retardation (IUGR), which presents as a failure of fetuses to achieve their intrinsic growth potential during pregnancy, has become one of the most common and challenging issues in animal production [1, 2]. IUGR alters the intestinal morphology, impairs intestinal barrier function, and creates feeding intolerance, all of which increase the occurrence of intestinal diseases and cause postnatal retardation of growth and development, resulting in high rates of mortality and morbidity [3–5]. IUGR neonates experience a high incidence of colitis since they show deficiencies in mucosal immunity and imbalances in T lymphocyte subpopulations and lack an efficient colonic barrier [6–9]. Therefore, protecting the colon against inflammation may be a pivotal requirement for the health of IUGR animals.

The colon is one site that is vulnerable to endoplasmic reticulum (ER) stress, a physiological response induced by protein aggregation or misfolding within the ER lumen [10]. A variety of physiological and pathological stressors, including bacteria and inflammation tone from the intestinal lumen and host, can induce colonic ER stress [11]. In addition, the highly secretory cells that occur in the colonic epithelium manufacture a range of complex proteins and antimicrobial molecules that are currently recognized as crucial targets for protein misfolding in the ER [12]. To counteract these negative effects, the conserved signal transduction pathways unfolded protein responses (UPR) have evolved. They are initially activated as adaptive responses that strengthen protein folding capacity, re-establish ER function, and restore intestinal barrier homeostasis [13]. However, once these adaptive mechanisms fail to resolve the folding defects, the UPR will switch to programmed cell death by triggering pro-apoptotic signaling cascades and even induce inflammatory responses [13, 14]. Indeed, ER stress has been identified as part of the intrinsic machinery of intestinal inflammation [11, 15].

Autophagy, a pro-survival mechanism for cells and organisms suffering from starvation or other diverse pathologies, plays an important physiological role in intestinal health [16]. Autophagy modulates the relationship between gut microflora and host immunity and thereby maintains intestinal barrier function, whereas impaired autophagy in intestinal epithelium can induce intestinal inflammation [17, 18]. Interestingly, autophagy is recognized as an emerging effector mechanism that regulates ER homeostasis [19]. The misfolded proteins and damaged ER fragments can be degraded and recycled by autophagy [19]. The UPR branches and UPR-associated transcription factors, such as activating transcription factor 4 (ATF4) and CCAAT/enhancer binding protein homologous protein (CHOP), have also been demonstrated to modulate the autophagic process [20]. Based on these findings, exploring the exact interaction between ER stress and autophagy in the context of inflammation may help to uncover the mechanism and identify prospective strategies for the IUGR-induced colon inflammation.

Stilbenes are a class of polyphenolic compounds that are widely distributed in blueberries, grapes, and other medicinal plants [21]. Their multiple biological activities, especially antioxidant and anti-inflammatory properties, suggest the potential value of stilbenes in the treatment or prevention of intestinal disorders [22–24]. Recently, considerable attention has focused on pterostilbene, a dimethylated analogue of resveratrol, due to its excellent metabolic stability, intestinal absorption features, and bioavailability [25, 26]. Our previous work indicated that pterostilbene had strong protective effects against intestinal barrier defects and small intestinal injuries in animals under the conditions of oxidative stress or immunological stress [27–29]. Pterostilbene was also found to mitigate colon inflammation in mice fed high-fat diets or treated with dextran sulfate sodium [30]. However, the potential for attenuation of the IUGR-induced colon inflammation by pterostilbene was not documented. Whether pterostilbene-mediated protection on intestine health is associated with its regulation of ER stress and/or autophagy is not known. The current study explored the mechanism by which pterostilbene protected against the IUGR-induced colon inflammation using a naturally occurring IUGR piglet model. We also revealed the crosstalk between ER stress and autophagy under inflammation conditions and the regulation of pterostilbene in these two events in the tumor necrosis factor-alpha (TNF-α)-treated human colonic epithelial cells (Caco-2).

## Materials and methods

### Reagents and antibodies

Pterostilbene was obtained from BOC Science (Shirley, NY, USA). TNF-α was purchased from Sino Biological, Inc. (Beijing, China). Tunicamycin, 4-phenylbutyric acid (4PBA), and FITC-dextran were purchased from Sigma-Aldrich (St. Louis, MO, USA). Rapamycin and 3-methyladenine (3MA) were purchased from CSNpharm (Arlington Heights, IL, USA). Chloroquine was purchased from MedChemExpress (Shanghai, China). Adenovirus expressing mCherry-GFP-LC3B fusion protein recognizing CD46 (AdPlus-mCherry-GFP-LC3B) was purchased from Beyotime (Haimen, Jiangsu, China). Dulbecco’s modified Eagle’s medium (DMEM), fetal bovine serum (FBS), and 1% penicillin and streptomycin were obtained from Thermo Fisher Scientific Inc. (Grand Island, NY, USA).

Antibodies used in Western blot analysis included: anti-occludin (1:3000; Proteintech; Chicago, IL, USA), anti-zonula occludens 1 (ZO-1; 1:1000; Proteintech), anti-glyceraldehyde phosphate dehydrogenase (GAPDH; 1:25,000; Proteintech), anti-nuclear factor kappa B p65 (NF-κB p65; 1:3000; Proteintech), anti-Lamin B1 (1:5000; Proteintech), anti-glucose-regulated protein 78 (GRP78; 1:3000; Proteintech), anti-CHOP (1:1000; Proteintech), anti-cleaved caspase 12 (c-Casp12; 1:2000; Bioss Biotechnology; Beijing, China), anti-phosphorylated protein kinase RNA-like ER kinase (PERK) (p-PERK; 1:2000; Affinity Biosciences; Cincinnati, OH, USA), anti-total-PERK (t-PERK; 1:1000; Affinity Biosciences), anti-phosphorylated inositol-requiring kinase 1 alpha (IRE1a) (p-IRE1a; 1:1000; Affinity Biosciences), anti-total-IRE1a (t-IRE1a; 1:2000; Affinity Biosciences), anti-activating transcription factor 6 (ATF6; 1:5000; Proteintech), anti-phosphorylated eukaryotic translational initiation factor 2 alpha (eIF2α) (p-eIF2α; 1:1000; Bioss Biotechnology), anti-total-eIF2α (t-eIF2α; 1:1000; Bioss Biotechnology), anti-Beclin1 (1:3000; Proteintech), anti-LC3 I/II (1:2000; Proteintech), anti-p62 (1:4000; Proteintech), anti-Rab7 (1:2000; Affinity Biosciences), and anti-lysosomal associated membrane protein 2 (LAMP2; 1:1000; Proteintech).

### Animals, experimental design, and sample collection

All animal experiments were reviewed and approved by the Institutional Animal Care and Use Committee of Nanjing Agricultural University (SYXK-2017-0027). Approximately 72 healthy sows (Landrace × Yorkshire) having same parity (second or third) and expected dates of confinement (< 4 d) were preselected during pregnancy. At delivery, only sows with about 11–13 live-born piglets were retained. The birth weights (BWs) of newborn piglets (Duroc × (Landrace × Yorkshire)) were recorded. Piglets were identified as normal birth weight (NBW) if their BWs were close to the mean litter BW (within 0.5 standard deviations), whereas those with BWs at least 2 standard deviations lower than the mean litter BW were defined as IUGR [27, 31]. All piglets were permitted to suckle naturally up to 21 days of age. After weaning, a total of 36 litters that met the selection criteria for NBW and IUGR piglets were reserved, and one IUGR and one NBW male piglet were selected from each litter. Half of the selected NBW and IUGR piglets received a basal diet (NBW-CON and IUGR-CON groups) for 14 d and the other half were fed a basal diet supplemented with 300 mg/kg pterostilbene (NBW-PTS and IUGR-PTS groups) over the same period. Each treatment group consisted of 6 replicate pens with 3 piglets per pen. The basal diet (Table S1) was formulated according to the nutrient requirements of swine (National Research Council, 2012) [32]. The level of supplemented pterostilbene was determined based on our previous publication [27, 28]. Diet and water were offered ad libitum during the entire experiment.

The diarrhea rates of piglets were determined as reported by Liu et al. [33]. At the endpoint of the experimental period, the piglets were weighed, and one piglet whose BW was close to the average weight of each replicate was chosen for sampling. Heparinized blood samples were withdrawn by anterior vena cava puncture, centrifuged at 2500 × *g* for 10 min at 4 °C, and stored at − 80 °C for analysis. The piglets were then euthanized, and the colon tissues were removed. After flushing with ice-cold PBS, approximately 1 cm of colon samples taken from the middle of the colon were immersed in 4% paraformaldehyde fixative solution for histological analysis. The colonic mucosa was scraped from the remainder with a glass microscope slide, snap-frozen in liquid nitrogen, and stored at − 80 °C for further analysis.

### Histopathological analysis

Colon tissues fixed in 4% paraformaldehyde were dehydrated in an ascending alcohol series, cleared with xylene, embedded in paraffin blocks, cut into 5 μm sections, and mounted on glass slides. After deparaffinizing and rehydrating, the sections were stained with hematoxylin and eosin (H&E) buffer, and the histologic alterations were observed using a light microscopy (Olympus Corp; Tokyo, Japan). The Chiu scoring system was employed to evaluate the colon mucosal injury. The colon goblet cell numbers were determined by incubating the slices with Alcian blue/periodic acid-Schiff stains as described previously [31]. The colon goblet cell density was calculated as the goblet cell count divided by the corresponding villus length.

### Plasma lipopolysaccharide (LPS) concentration

Plasma LPS levels were measured with an enzyme-linked immunosorbent assay (ELISA) kit obtained from CUSABIO Biotech (Wuhan, Hubei, China).

### Analysis of mucosal immune status

The colon mucosa was homogenized and centrifuged at 5000 × *g* for 5 min, and the concentrations of TNF-α, interleukin (IL)-1β, IL-4, IL-10, mucin 2, and trefoil factor 3 (TFF3) of the supernatants were determined with porcine-specific ELISA kits (CUSABIO Biotech). The colon myeloperoxidase (MPO) activity was measured with a commercial kit (Jiancheng Bioengineering Institute; Nanjing, Jiangsu, China). The protein levels of the supernatants were quantified using a bicinchoninic acid protein assay kit (Beyotime) for normalization of these parameters.

### Cell culture, treatment, and cell viability analysis

Caco-2 cell line was kindly gifted by Dr. Xiang Hou (Jiangsu Academy of Agricultural Sciences; Nanjing, Jiangsu, China). Caco-2 cells at passage numbers 28–36 were grown in DMEM medium supplemented with 10% FBS and 1% penicillin and streptomycin at 37 °C in a humidified atmosphere containing 5% CO_2_. The optimal treatment concentrations of TNF-α and pterostilbene were determined by exposing Caco-2 cells to TNF-α (0–50 ng/mL) with or without pterostilbene (0–250 μmol/L) for 24 h. ER stress in Caco-2 cells was induced and inhibited by tunicamycin (0.5 μg/mL for 24 h) and 4PBA (1 mmol/L for 24 h), respectively. Rapamycin (1 μmol/L for 24 h) and 3MA (5 μmol/L for 24 h) were used to induce and inhibit autophagy in Caco-2 cells, respectively. Chloroquine (20 μmol/L for 24 h) was used to obstruct the fusion of autophagosomes and lysosomes in Caco-2 cells.

The Cell Counting Kit-8 (CCK-8; Dojindo Molecular Technologies; Shanghai, China) was used to evaluate the viability of Caco-2 cells. Briefly, Caco-2 cells were seeded in 96-well microplates at a density of 1 × 10^4^ cells/well. After 24 h, Caco-2 cells were treated with a range of TNF-α or pterostilbene for 24 h. Then, 10 μL CCK-8 reagent was added to each well for 1.5 h, and the absorbance was measured by a microplate reader (Thermo Fisher Scientific Inc.) at 450 nm.

### Analysis of autophagic flux

The autophagic flux was monitored by transfecting Caco-2 cells with AdPlus-mCherry-GFP-LC3B. The Caco-2 cells were seeded in confocal dishes at a density of 2 × 10^5^ cells/well until confluence reached 50%. After infection with AdPlus-mCherry-GFP-LC3B dissolved in a complete DMEM medium (multiplicity of infection = 80) for 24 h, the cells were co-incubated with TNF-α and tunicamycin, 4PBA, rapamycin, or pterostilbene for another 24 h. The expression of GFP and mCherry was visualized and captured with a laser scanning confocal microscope (Carl Zeiss; Oberkochen, Germany).

### The permeability of Caco-2 cell monolayers

Caco-2 cells (2 × 10^5^ cells/well) were grown in 12-well permeable culture chambers (12 mm diameter inserts and 0.4 μm pore size) with a complete DMEM medium until they formed a tight monolayer with a transepithelial electrical resistance (TER) of 500 Ω. Thereafter, the cells were subjected to various treatments, and the TER and FITC-dextran flux were analyzed to determine the permeability of Caco-2 cell monolayers. The TER of each culture chamber was tested at 3 different sites using the Millicell-ERS resistance system (Millipore; Bedford, MA, USA) and corrected against the blank control. The net resistance was multiplied by the membrane area to give the resistance in Ω cm^2^. For FITC-dextran flux analysis, a complete DMEM medium containing 500 μg/mL FITC-dextran was placed in the upper chamber of the transwells. The FITC-dextran flux was measured by collecting the basal medium every 30 min for 3 h with replacement of the sampled volume with fresh medium without FITC-dextran at each sampling time and expressed as the flux into the basal chamber as a percentage of the total FITC-dextran initially added to the apical chamber.

### Cytokine concentrations in the supernatants of Caco-2 cells

Caco-2 cells were seeded in 6-well plates at a density of 5 × 10^5^ cells/well for 24 h and then exposed to TNF-α and pterostilbene with or without tunicamycin and chloroquine for 24 h. The supernatants of Caco-2 cells were collected to determine the contents of IL-1β and IL-6 with ELISA kits (Multi Sciences Biotech; Hangzhou, Zhejiang, China). All procedures strictly followed the manufacturer’s protocols.

### Apoptosis measurement

Terminal deoxynucleotidyl transferase dUTP nick end labeling (TUNEL) staining and flow cytometry assays were carried out to determine the apoptotic index of the colon tissues and Caco-2 cells, respectively. The methods can be found in our previous study [28].

### Immunofluorescent staining

Caco-2 cells were plated in 12-well plates at a density of 2 × 10^5^ cells/well for 24 h and then subjected to the study treatments. Subsequently, the cells were fixed in 4% paraformaldehyde, permeabilized with 0.1% Triton X-100, and incubated with 3% bovine serum albumin (BSA). The cells were then incubated overnight with the primary body against NF-κB p65 (1:200) at 4 °C, followed by Alexa Fluor 594-conjugated secondary antibody (1:200) for 1 h and 2-(4-amidinophenyl)-6-indolecarbamidine dihydrochloride (DAPI) for 1 min at room temperature. Images were recorded with a confocal laser scanning microscope (Carl Zeiss).

### Total RNA isolation and quantitative real-time PCR (qRT-PCR) analysis

All reagents were purchased from Vazyme (Nanjing, Jiangsu, China). Total RNA from colon samples and Caco-2 cells was isolated with the FastPure Cell/Tissue Total RNA Isolation Kit and reverse-transcribed into cDNAs with the HiScript III 1st Strand cDNA Synthesis Kit. The ChamQ SYBR qPCR Master Mix and the StepOnePlus PCR System (Applied Biosystems; Carlsbad, CA, USA) were used to detect the expression levels of target genes by qRT-PCR analysis. Thermal cycling was initiated at 95 °C for 30 s, followed by 40 cycles of 95 °C for 10 s and 60 °C for 30 s. The specific primers for *GRP78*, glucose-regulated protein 94 (*GRP94*), *CHOP*, *ATF4*, spliced X-box binding protein-1 (*sXBP-1*), *TNF-α*, *IL-1β*, *IL-6*, and *GAPDH* were obtained from Sangon Biotech (Shanghai, China). The primer sequences were provided in Table S2. The results were calculated using the 2^−ΔΔCT^ method and normalized to *GAPDH*.

### Analysis of *sXBP-1* mRNA in Caco-2 cells

Real-time PCR with T100 Thermal Cycler (Bio-Rad; Hercules, CA, USA) was carried out to determine the mRNA levels of *sXBP-1* in Caco-2 cells. The PCR products were separated on 1.8% agarose gel and visualized with GelRed DNA stain. The primer sequences can be found in Table S2.

### Western blot analysis

All reagents for protein extraction and determination of protein concentration were obtained from Beyotime. Total protein from colon samples and Caco-2 cells was extracted using the lysis buffer containing a cocktail of phosphatase and protease inhibitors. A nuclear protein extraction kit was employed to isolate the nuclear protein in colon tissues and Caco-2 cells. After measurement of the protein contents, 30 μg of protein from each sample was separated by SDS-PAGE and electro-transferred to the polyvinylidene fluoride membranes. Subsequently, the membranes were blocked with 5% BSA for 1 h at room temperature and then incubated with specific primary antibodies overnight at 4 °C with gentle rocking. After washing with the Tris-buffered saline containing 1% Tween 20, the corresponding secondary antibodies were added and incubated for 1.5 h at room temperature. Immunoreactive bands were captured by the ChemiDoc™ imaging system (Bio-Rad) and analyzed using the Gel-Pro Analyzer software.

### Statistical analysis

All statistical analyses were performed using SPSS software version 26 (Chicago, IL, USA). Data from at least three independent experiments were shown as means with their standard errors (SE). For comparison of multiple datasets, one-way analysis of variance (ANOVA) with Tukey’s multiple-range test was employed. Values with *P* < 0.05 were considered statistically significant.

## Results

### Pterostilbene decreases diarrhea rates and improves colonic barrier function of the IUGR piglets

**Fig. 1:**
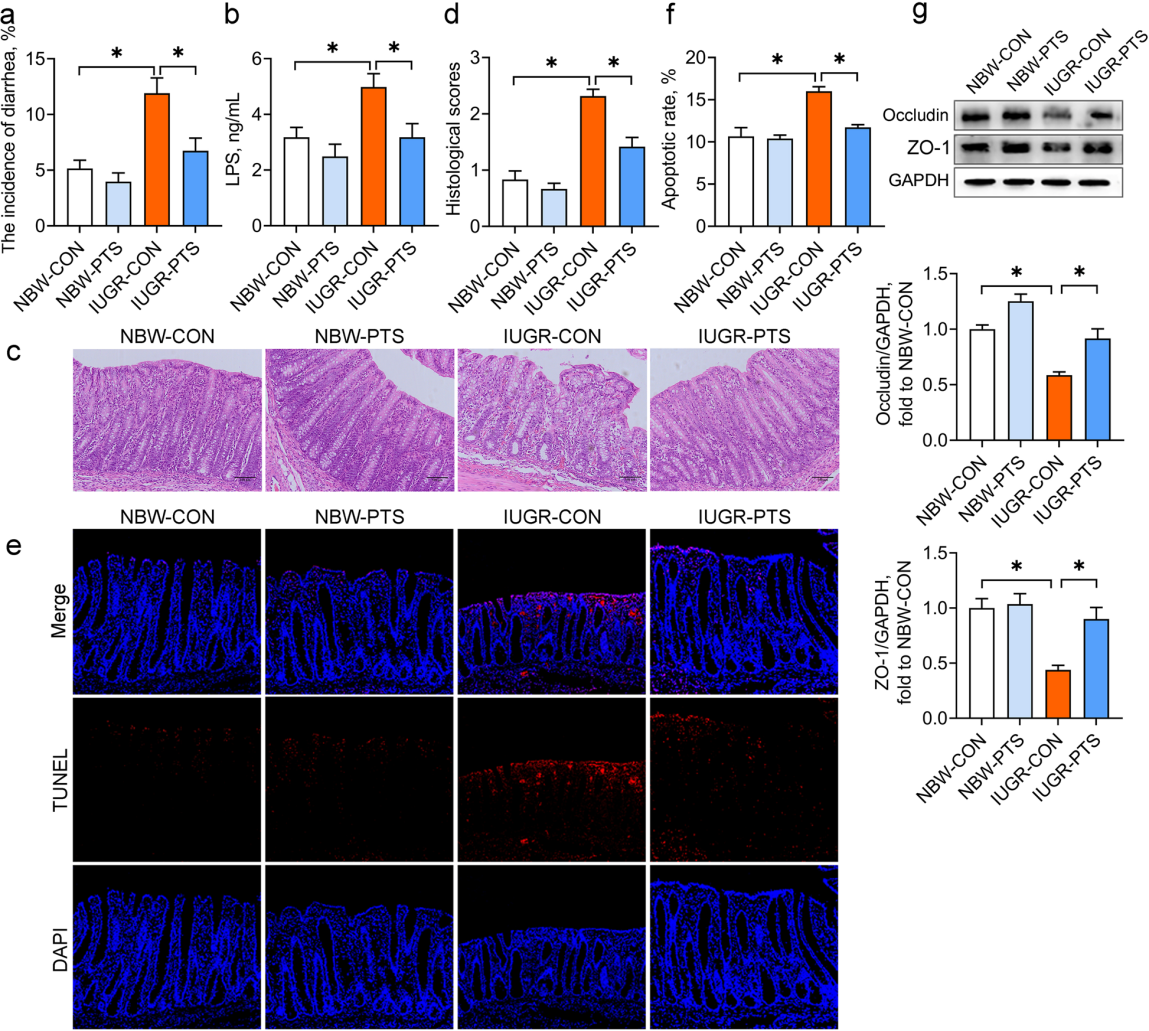
Pterostilbene decreases diarrhea rates and improves colonic barrier function of the IUGR piglets. **a** The diarrhea rates of piglets; **b** Plasma LPS concentration; **c** Representative photomicrographs of hematoxylin and eosin-stained colonic sections from piglets; **d** Histologic scores were determined using the Chiu scoring system; **e** Representative photomicrographs of terminal deoxynucleotidyl transferase dUTP nick end labeling (TUNEL)-stained colonic sections from piglets; **f** Quantitative analysis of TUNEL-positive cells in the colon; **g** The protein levels of colon tight junction proteins were determined by Western blot analysis. Data from at least three independent experiments were presented as mean ± SE (*n* = 6). ^*^P < 0.05

To evaluate the protective potential of pterostilbene on intestinal health of the IUGR piglets, we recorded the diarrhea rates and determined the contents of circulating LPS, a bacterially generated endotoxin that penetrates the intestinal epithelial barrier and reaches the bloodstream when the intestinal permeability is increased [34]. As shown in Fig. 1a and b, the diarrhea rates and plasma LPS levels were significantly higher in the IUGR piglets than in their NBW counterparts, whereas these negative effects caused by IUGR were attenuated after pterostilbene treatment (*P* < 0.05). We also assessed the colon morphology, apoptosis, and tight junction protein expression of piglets to determine the effects of pterostilbene on colon barrier function. H&E staining indicated extensive damage to the colon epithelium of IUGR piglets, with injuries including hemorrhage, crypt abscesses, and lymphocyte infiltration to the lamina propria (Fig. 1c). The histological scores and apoptotic rates were also higher in the colon of the IUGR piglets than in those of the NBW piglets (*P* < 0.05; Fig. 1d-f). Moreover, IUGR downregulated colon protein levels of occludin and ZO-1 of piglets (*P* < 0.05; Fig. 1g). In contrast, dietary pterostilbene administration improved the colon architecture and blocked hemorrhage and lymphocyte infiltration to the lamina propria, thereby reducing the histological scores of the IUGR colon (*P* < 0.05). The increased number of TUNEL-positive cells and the reduced protein levels of occludin and ZO-1 in the IUGR colon were also mitigated by pterostilbene (*P* < 0.05). These data suggest that pterostilbene potentially maintains colonic barrier function of the IUGR piglets.

### Pterostilbene alleviates inflammatory responses and regulates immune function in the IUGR colon

**Fig. 2:**
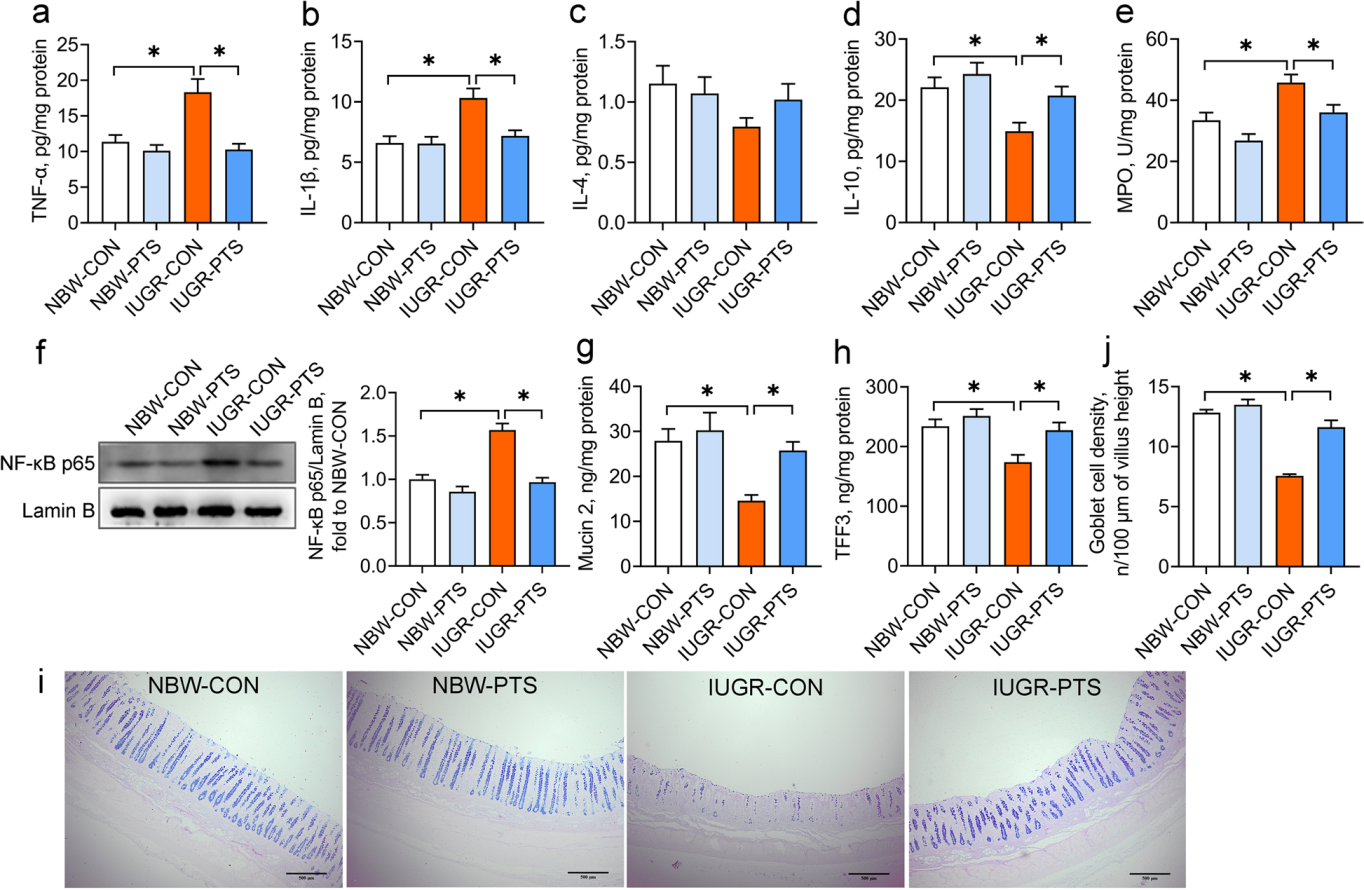
Pterostilbene alleviates inflammatory responses and regulates immune function in the IUGR colon. **a**-**d** The contents of TNF-α, IL-1β, IL-4, and IL-10 in the colon were measured by ELISA assays; **e** Colon myeloperoxidase (MPO) activity; **f** Colon protein levels of nuclear NF-κB p65 were determined by Western blot analysis; **g** Colonic mucin 2 levels; **h** Colonic trefoil factor 3 (TFF3) levels; **i** Representative micrographs of Alcian blue/periodic acid-Schiff stained colonic sections from piglets; **j** Goblet cell density of colon. Data from at least three independent experiments were presented as mean ± SE (*n* = 6). ^*^P < 0.05

The possible inhibition of the IUGR-induced colon inflammation by pterostilbene was evaluated by measurement of several markers of inflammatory responses. Compared with the NBW piglets, the IUGR piglets had higher concentrations of TNF-α and IL-1β but lower concentrations of IL-10 in the colon (*P* < 0.05; Fig. 2a-d). The MPO activity and nuclear NF-κB p65 protein expression in the colon were also increased by IUGR (*P* < 0.05; Fig. 2e and f). Further assessment of colon immune function revealed marked reductions in the contents of mucin 2 and TFF3 and the number of goblet cells in the IUGR piglets compared to their NBW counterparts (*P* < 0.05; Fig. 2g-j). Conversely, treatment with pterostilbene decreased the concentrations of TNF-α and IL-1β, the MPO activity, and the translocation of NF-κB p65 from the cytoplasm to the nucleus, while increasing the IL-10 levels in the IUGR colon (*P* < 0.05). Pterostilbene also promoted the secretion of mucin 2 and TFF3 and increased the number of goblet cells in the IUGR colon (*P* < 0.05). These observations indicate that pterostilbene suppresses inflammatory responses and overcomes defective immune function in the IUGR colon.

### Pterostilbene inhibits ER stress and restores autophagic flux in the IUGR colon

**Fig. 3:**
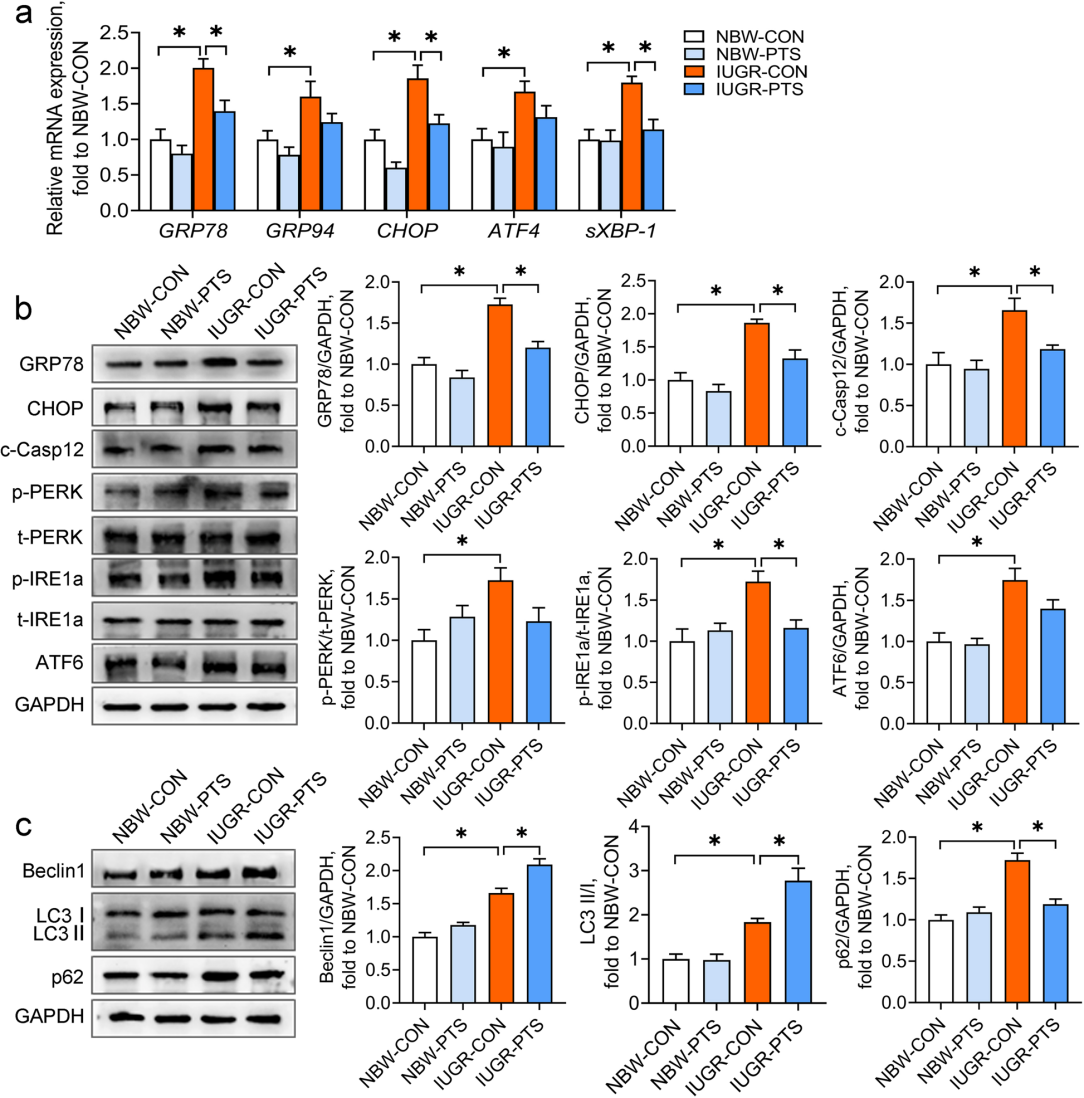
Pterostilbene inhibits ER stress and restores autophagic flux in the IUGR colon. **a** qRT-PCR analysis was performed to determine the mRNA abundance of *GRP78*, *GRP94*, *CHOP*, *ATF4*, and *sXBP-1* in the colon; **b**, **c** The protein levels of ER stress markers, UPR sensors, and autophagy-associated molecules were determined by Western blot analysis. Data from at least three independent experiments were presented as mean ± SE (*n* = 6). ^*^P < 0.05

ER stress and autophagy are the crucial mechanisms implicated in inflammatory bowel diseases [14, 16]. However, an association between ER stress or autophagy and the IUGR-induced colon inflammation remains unconfirmed. Our results suggested that IUGR piglets showed apparent ER stress in the colon, as indicated by the increases in the mRNA abundance of *GRP78*, *GRP94*, *CHOP*, *ATF4*, and *sXBP-1* and the protein levels of GRP78, CHOP, and c-Casp12 (*P* < 0.05; Fig. 3a and b). We also determined the expression of the three arms of the UPR (i.e., IRE1, PERK, and ATF6) and found that IUGR induced the phosphorylation of PERK and IRE1a and elevated ATF6 protein expression in the colon (*P* < 0.05). In contrast, pterostilbene treatment significantly downregulated the mRNA expression of *GRP78*, *CHOP*, and *sXBP-1* and the protein expression of GRP78, CHOP, and c-Casp12 in the IUGR colon (*P* < 0.05). The phosphorylation of IRE1a in the IUGR colon was also significantly inhibited by pterostilbene (*P* < 0.05).

The measurement of autophagy-associated proteins in the colon showed that the protein levels of Beclin1 and p62 and the ratio of LC3 II/I were higher in the IUGR piglets than in the NBW piglets (*P* < 0.05; Fig. 3c), indicating that IUGR may disturb the process of autophagosomal degradation in the colon. Pterostilbene treatment further upregulated the protein expression of Beclin1 and the ratio of LC3 II/I but markedly suppressed the protein expression of p62 in the IUGR colon (*P* < 0.05). To summarize, these results suggest that pterostilbene inhibits colon ER stress and reverses impaired autophagic flux in the IUGR colon.

### The crosstalk between ER stress and autophagy in the TNF-α-exposed Caco-2 cells

**Fig. 4:**
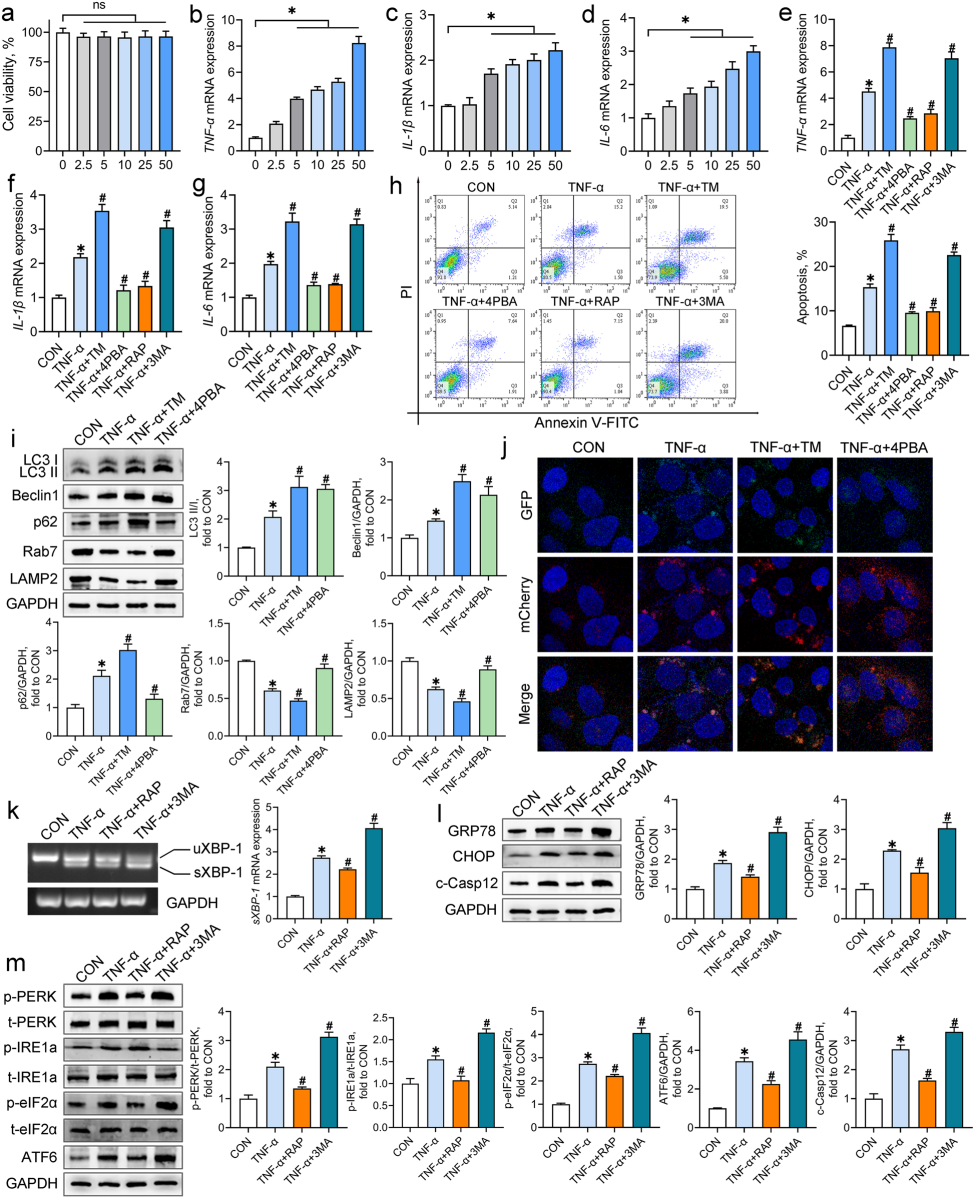
The crosstalk between ER stress and autophagy in the TNF-α-exposed Caco-2 cells. Caco-2 cells were incubated with TNF-α (0–50 ng/mL) for 24 h. **a** The cell viability was measured by the CCK-8 assay (*n* = 6); **b**-**d** The mRNA abundance of inflammatory cytokines was measured by qRT-PCR analysis (*n* = 4). Caco-2 cells were co-incubated with TNF-α and tunicamycin (TM), 4-phenylbutyric acid (4PBA), rapamycin (RAP), or 3-methyladenine (3MA). **e**-**g** The mRNA abundance of inflammatory cytokines was measured by qRT-PCR analysis (*n* = 4); **h** Representative scatter plots and quantitative analysis of apoptotic cells in experimental groups as analyzed by the flow cytometry (*n* = 4); **i** Western blot analysis was conducted to determine the protein levels of autophagy-associated molecules (*n* = 4); **j** Autophagic flux of Caco-2 cells was analyzed through transfection with AdPlus-mCherry-GFP-LC3B. The representative immunofluorescent photographs were indicated. GFP dots are green. mCherry dots are red (*n* = 4); **k** RT-PCR assay was conducted to detect the mRNA expression of *sXBP-1* in Caco-2 cells (*n* = 4); **l**, **m** Western blot analysis was carried out to detect the protein levels of ER stress markers and UPR sensors in Caco-2 cells (*n* = 4). Data from at least three independent experiments were presented as mean ± SE. ns means no significance; ^*^P < 0.05 vs. CON group; ^#^*P* < 0.05 vs. TNF-α group

Having determined the involvement of ER stress and autophagy in the IUGR-induced colon inflammation, we investigated the potential crosstalk between these in the context of inflammation using an in vitro system, the Caco-2 cell line. CCK-8 and qRT-PCR assays were conducted to assess the effects of TNF-α on the cell viability and inflammatory responses in Caco-2 cells, respectively. TNF-α stimulation at up to 50 ng/mL developed no obvious differences in cell viability (*P* > 0.05) but a concentration-dependent upregulation (5–50 ng/mL) of the mRNA abundance of *TNF-α*, *IL-1β*, and *IL-6* (*P* < 0.05) in Caco-2 cells (Fig. 4a-d). Thus, an intermediate dosage of TNF-α (10 ng/mL) was chosen to induce inflammation in Caco-2 cells.

To assess the effects of ER stress and autophagy on inflammatory responses and cell apoptosis, Caco-2 cells were co-incubated with TNF-α and the inducers or inhibitors of ER stress and autophagy. Treatment with the ER stress inducer tunicamycin and the autophagy inhibitor 3MA aggravated the TNF-α-induced increases in the mRNA abundance of *TNF-α*, *IL-1β*, and *IL-6*, and cell apoptosis in Caco-2 cells, whereas the ER stress inhibitor 4PBA and the autophagy inducer rapamycin had reversed actions (*P* < 0.05; Fig. 4e-h).

To clarify the regulation of ER stress on autophagy activity under inflammation conditions, autophagy-related proteins and autophagic flux in the TNF-α-treated Caco-2 cells were determined after the combined treatment with tunicamycin or 4PBA. TNF-α treatment notably increased the ratio of LC3 II/I and the protein expression of Beclin1 and p62 but reduced the protein expression of Rab7 and LAMP2 (*P* < 0.05; Fig. 4i). All of these changes were further aggravated by the combined treatment with tunicamycin (*P* < 0.05). Although the ratio of LC3 II/I and Beclin1 protein in the TNF-α-exposed cells were upregulated by 4PBA, the protein levels of p62, Rab7, and LAMP2 in the TNF-α-exposed cells were recovered to the control levels by 4PBA (*P* < 0.05). The analysis of autophagic flux of Caco-2 cells illustrated basal autophagy in the control cells, which displayed only weak GFP and mCherry signals (Fig. 4j). TNF-α exposure induced the accumulation of autophagosomes (yellow puncta merged by mCherry and GFP fluorescence) and few autolysosomes (mCherry puncta) in Caco-2 cells, suggesting a blockage of autophagosome clearance. Treatment with tunicamycin further increased the number of autophagosomes but did not alter the number of autolysosomes in the TNF-α-exposed cells. In contrast, 4PBA treatment of the TNF-α-treated cells increased the number of autolysosomes but reduced the number of autophagosomes. These results suggest that the induction of ER stress worsens the TNF-α-induced obstruction of autophagic flux in Caco-2 cells, whereas the inhibition of ER stress exerts opposite effects.

**Fig. 5:**
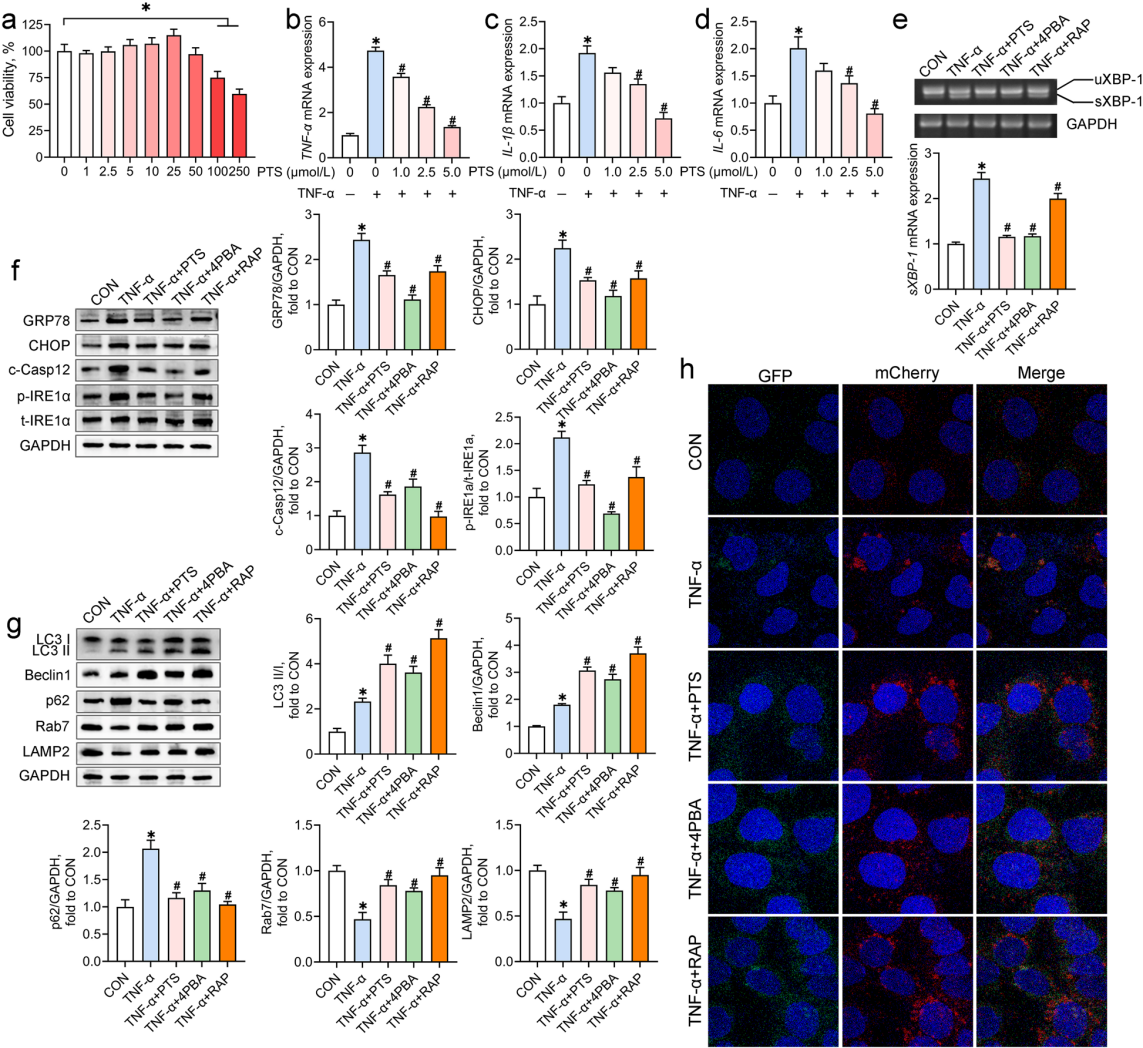
Pterostilbene inhibits ER stress and promotes autophagic flux in the TNF-α-treated Caco-2 cells. **a** Caco-2 cells were incubated with various concentrations of pterostilbene for 24 h and the cell viability was detected by the CCK8 assay (*n* = 6); **b**-**d** Caco-2 cells were co-incubated with TNF-α (10 ng/mL) and pterostilbene (0–5 μmol/L) for 24 h and the mRNA abundance of inflammatory cytokines was measured by qRT-PCR analysis (*n* = 4). Caco-2 cells were co-incubated with TNF-α and pterostilbene, 4-phenylbutyric acid (4PBA), or rapamycin (RAP) for 24 h. **e** RT-PCR assay was conducted to detect the mRNA level of *sXBP-1* in Caco-2 cells (*n* = 4); **f**, **g** Western blot analysis was carried out to detect the protein levels of ER stress markers, UPR sensors, and autophagy-associated proteins in Caco-2 cells (*n* = 4); **h** Autophagic flux of Caco-2 cells was analyzed through transfection with AdPlus-mCherry-GFP-LC3B. The representative immunofluorescent photographs were indicated. GFP dots are green. mCherry dots are red (*n* = 4). Data from at least three independent experiments were presented as mean ± SE. ^*^P < 0.05 vs. CON group; ^#^*P* < 0.05 vs. TNF-α group

The effects of autophagy on ER homeostasis in the context of inflammation were ascertained by measurement of the ER stress markers and the UPR sensors. The mRNA levels of *sXBP-1* and the protein levels of GRP78, CHOP, and c-Casp12 in Caco-2 cells were increased by TNF-α treatment (*P* < 0.05; Fig. 4k and l). TNF-α exposure also increased the phosphorylation of PERK, IRE1a, and, eIF2α, and the protein expression of ATF6 in Caco-2 cells (*P* < 0.05; Fig. 5m). Treatment with 3MA aggravated the TNF-α-induced elevations in ER stress markers and UPR sensors; however, rapamycin treatment mitigated these negative effects observed following TNF-α treatment (*P* < 0.05).

### Pterostilbene inhibits ER stress and promotes autophagic flux in the TNF-α-treated Caco-2 cells

We next assessed the effects of pterostilbene on ER stress and autophagy activity in the TNF-α-treated Caco-2 cells with 4PBA and rapamycin as positive controls. The cell viability assay was first conducted to determine the effect of pterostilbene on cell viability in Caco-2 cells. Compared with the untreated cells, treatment with pterostilbene at increasing concentrations (0–50 μmol/L) showed no significant cytotoxicity in Caco-2 cells (*P* > 0.05; Fig. 5a). Thus, lower than 50 μmol/L pterostilbene was selected to determine the anti-inflammatory action of pterostilbene in the TNF-α-treated Caco-2 cells. As shown in Fig. 5b-d, pterostilbene (0–5 μmol/L) dose-dependently blocked the TNF-α-induced elevation of the mRNA abundance of *TNF-α*, *IL-1β*, and *IL-6* in Caco-2 cells (*P* < 0.05). Therefore, 2.5 μmol/L of pterostilbene was chosen for subsequent in vitro studies.

As indicated in Fig. 5e and f, pterostilbene was as effective as 4PBA at suppressing the splicing of *XBP-1* mRNA and downregulating the protein levels of GRP78, CHOP, c-Casp12, and phosphorylated IRE1a in the TNF-α-exposed cells (*P* < 0.05). In addition, treatment with pterostilbene had similar effects on autophagy activity to those seen with rapamycin, as pterostilbene increased the ratio of LC3 II/I and the protein levels of Beclin1, Rab7, and LAMP2 and decreased p62 protein in the TNF-α-exposed cells (*P* < 0.05; Fig. 5g). Moreover, pterostilbene promoted the formation autolysosomes and consumption of autophagosomes in the TNF-α-exposed cells (*P* < 0.05; Fig. 5h). Taken together, these findings indicate that pterostilbene mitigates ER stress and accelerates autophagic flux in the TNF-α-treated cells.

### Pterostilbene alleviates the TNF-α-induced colon inflammation by inhibiting ER stress and promoting autophagic flux

**Fig. 6:**
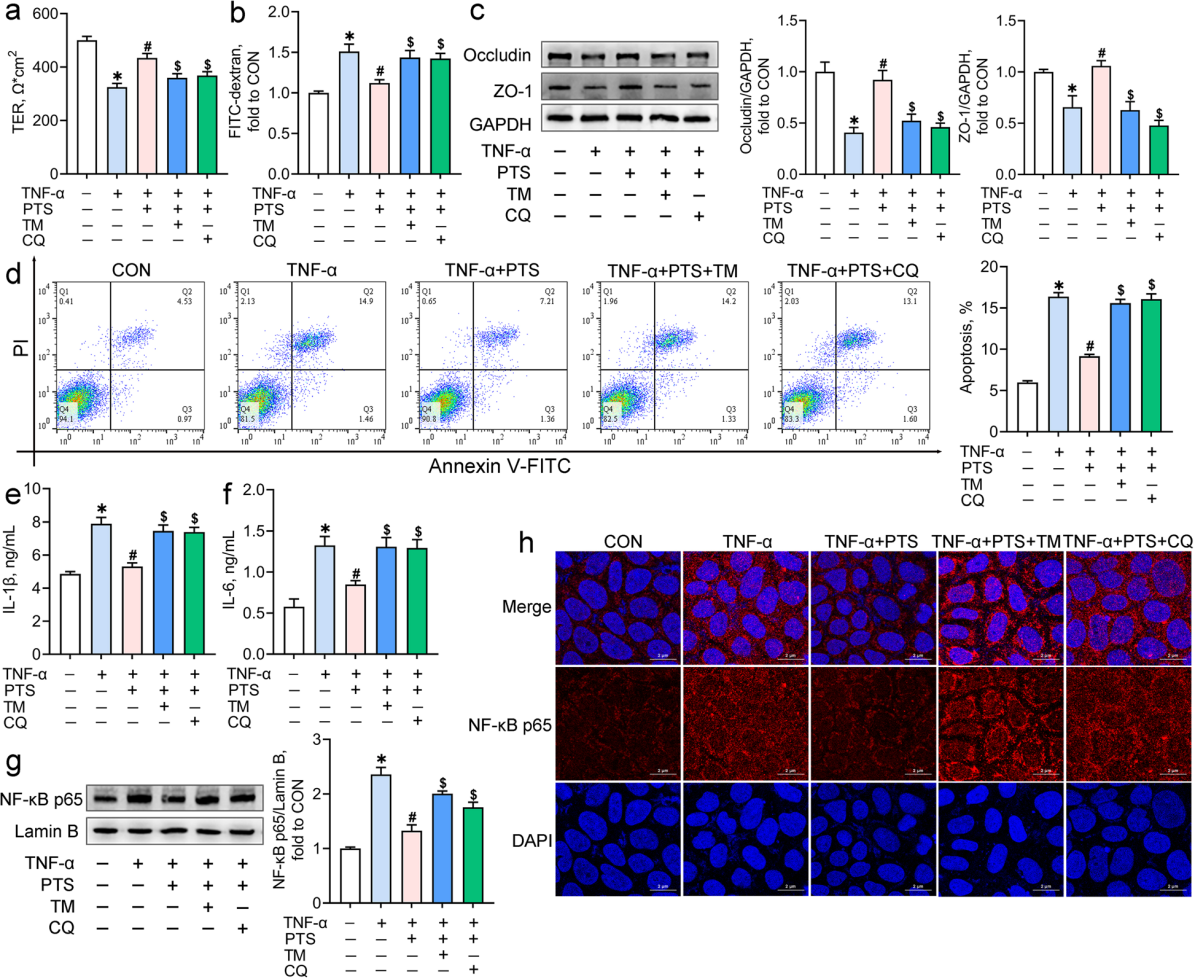
Pterostilbene alleviates the TNF-α-induced inflammation and barrier dysfunction by inhibiting ER stress and promoting autophagic flux. Caco-2 cells co-incubated with TNF-α and pterostilbene were treated with tunicamycin (TM) or chloroquine (CQ) for 24 h. The transepithelial electrical resistance (**a**) and FITC-dextran flux (**b**) of Caco-2 cell monolayers were determined; **c** Western blot analysis was conducted to determine the protein levels of intestinal tight junction complexes in Caco-2 cells; **d** Representative scatter plots and quantitative analysis of apoptotic cells in various treatments as analyzed by the flow cytometry; **e**, **f** ELISA assays were carried out to determine the contents of IL-1β and IL-6 in the supernatants of Caco-2 cells; **g** Nuclear NF-κB p65 protein levels in Caco-2 cells were detected by Western blot analysis; **h** Immunofluorescence staining of NF-κB p65 in Caco-2 cells. The red and blue fluorescence represent the NF-κB p65 protein and nucleus, respectively. Data from at least three independent experiments were presented as mean ± SE (*n* = 4). ^*^P < 0.05 vs. CON group, ^#^*P* < 0.05 vs. TNF-α group, ^$^*P* < 0.05 vs. TNF-α + PTS group

To investigate the roles of ER stress and autophagic flux in pterostilbene-mediated protection on colonic inflammation, Caco-2 cells co-incubated with TNF-α and pterostilbene were treated with the ER stress inducer tunicamycin or the autophagosome-lysosome binding inhibitor chloroquine. Treatment with pterostilbene improved the TNF-α-induced intestinal barrier dysfunction in Caco-2 cells, as indicated by the increases in TER and the protein levels of occludin and ZO-1 and the decrease in FITC-dextran flux (*P* < 0.05; Fig. 6a-c). Pterostilbene also significantly inhibited cell apoptosis, the release of IL-1β and IL-6, and the translocation of NF-κB p65 from the cytoplasm to the nucleus in the TNF-α-treated cells (*P* < 0.05; Fig. 6d-h). However, both tunicamycin and chloroquine counteracted the pterostilbene-mediated beneficial effects on intestinal barrier function, cell apoptosis, and inflammatory responses in the TNF-α-treated Caco-2 cells (*P* < 0.05), suggesting that the attenuation of pterostilbene on inflammatory response and barrier dysfunction depends on the inhibition of ER stress and the promotion of autophagosome-lysosome fusion.

## Discussion

ER stress and autophagy have been recognized as the crucial mechanisms involved in inflammatory bowel disease [12, 17, 18]; however, a role in IUGR-induced colon inflammation has not been established. The present study revealed that IUGR caused the upregulation of ER stress indicators (GRP78, CHOP, and c-Casp12) and the activation of UPR sensors (IRE1a, PERK, and ATF6), and altered the expression of the proteins responsible for the formation and degradation of autophagosomes (LC3 II, Beclin1, and p62) in the colon. These changes implicated ER stress and impaired autophagy in the IUGR-induced colon inflammation. In addition, our investigation of the crosstalk between ER stress and autophagy in the TNF-α-treated Caco-2 cells indicated that the autophagy inhibitor 3MA intensified the TNF-α-induced ER stress, and the ER stress inducer tunicamycin, in turn, augmented the abnormal accumulation of impaired autophagosomes caused by TNF-α. In particular, tunicamycin and 3MA exacerbated the TNF-α-induced inflammatory responses and apoptosis in Caco-2 cells, whereas the ER stress inhibitor 4PBA and the autophagy activator rapamycin had opposite effects. These data imply that the regulation of ER stress and autophagy may be the potential therapeutic approaches for the IUGR-induced colon inflammation.

Pterostilbene is a naturally occurring stilbene with various biofunctionalities and its effects on animal growth performance have been investigated [29, 35, 36]. We previously reported that pterostilbene could act as a feed additive in the broiler diet to prevent the growth performance descent caused by immunological stress and oxidative stress [29, 35]. Pterostilbene (300 mg/kg) also afforded protection against the diquat injection-induced body weight loss of piglets [36]. In this research, however, the compromised growth performance, including body weight, average daily gain, and average daily feed intake, of the IUGR piglets was not significantly improved by pterostilbene (300 mg/kg; data no shown). These conflicting findings are probably associated with the physiological conditions of animals.

Although the growth performance of the IUGR piglets was not significantly altered, pterostilbene induced marked reductions in diarrhea rates and colon inflammation of the IUGR piglets. These benefits may result from the potent anti-inflammation action of pterostilbene. The available evidence from in vivo and in vitro experiments has indicated that pterostilbene prevents inflammatory responses by suppressing NF-κB signals [37–39]. Consistent with those findings, we observed that pterostilbene significantly downregulated the expression of pro-inflammatory mediators and prevented the nuclear accumulation of NF-κB p65 in the IUGR piglet colons and the TNF-α-treated Caco-2 cells.

In addition, the pterostilbene-mediated protection against ER stress may provide another explanation for the attenuation of the IUGR-induced colon inflammation. IRE1, a conserved ER transmembrane protein, can be activated when the ER homeostasis is perturbed. Notably, the kinase domain of IRE1 complexes with the IkB kinase via interaction with TNF-receptor activating factor 2, which expedites the degradation of IkBα and triggers NF-κB signals, resulting in inflammatory responses [40]. A previous study with HK-2 cells has reported that resveratrol, the parent compound of pterostilbene, prevented the LPS- and tunicamycin-induced overproduction of inflammatory factors and NF-κB activation by inactivation of IRE1 [41]. Liu et al. [42] have also verified that the knockdown of IRE1 had parallel therapeutic effects to those seen with pterostilbene on the TNF-α-induced inflammatory responses in endothelial cells. In this study, pterostilbene treatment decreased the phosphorylation of IRE1a in both the IUGR colon and the TNF-α-treated Caco-2 cells, which may further inhibit the nuclear translocation of NF-κB p65 and the release of inflammatory factors. Intriguingly, the beneficial roles of pterostilbene in inactivation of NF-κB signals and downregulation of inflammatory cytokines in the TNF-α-treated Caco-2 cells were abrogated under a combined treatment with the ER stress inducer tunicamycin. These data suggest the protective effects of pterostilbene on colon inflammation depend on its suppression of ER stress.

Autophagy is an indispensable pro-survival process under ER stress conditions that allows the misfolded proteins and damaged cellular components to undergo lysosome-dependent self-digestion and recycling [16]. Also, autophagy is strictly interconnected with inflammatory responses, as the damaged and non-functional mitochondria and aggregated inflammasome structures mainly removed through the autophagic process are the crucial mediators for inflammasome activation [43, 44]. Wang et al. [45] have demonstrated that pterostilbene prevented the activation of NLR family, pyrin domain containing three inflammasome (NLRP3) by inducing autophagy in immortalized rat kidney proximal tubular epithelial cells. In the current study, pterostilbene promoted the protein expression of LC3 II and Beclin1 and recovered the protein levels of Rab7 and LAMP2 in the TNF-α-treated Caco-2 cells, which not only reveals the strong regulation of pterostilbene in autophagy but also provides a possible mechanism by which pterostilbene alleviates the TNF-α-induced inflammatory responses in Caco-2 cells.

Notably, autophagy is a dynamic biological process involving autophagosome formation, autophagosome-lysosome fusion, and final degradation. Disruption of any one of these steps may invoke dysregulated autophagy and cause damage to cells and tissues [16]. LC3 is a protein located in the autophagosomal inner membrane and it, along with Beclin1 protein, plays a pivotal role in the formation of autophagosomes [46]. The elevations in LC3 II in a stable state may result from autophagy activation or downstream blockage of autophagic vacuole processing. P62 is an autophagy substrate, and its reduction is associated with the promotion of autolysosome degradation [47]. Therefore, pterostilbene-mediated increases in the protein levels of LC3 II and Beclin1 and decrease in p62 protein in the IUGR colon and the TNF-α-exposed Caco-2 cells confirmed that pterostilbene enhanced autophagic activity and accelerated autophagic flux in this study.

Emerging evidence has indicated that the protective effects of natural phytochemicals on autophagy are related to their regulation of the fusion of lysosomes with mature autophagosomes, a crucial step for the degradation of autophagic cargo [48–50]. Wang et al. [50] have reported that resveratrol mitigated oxidative stress-induced autophagic dysfunction by promoting the expression of autophagosome-lysosome fusion-promoting protein Rab7. Pterostilbene was also demonstrated to restore impaired autophagic flux and prevent acetaminophen-induced liver injury [51]. However, the protective responses were abolished by treatment with chloroquine, the autophagosome-lysosome binding inhibitor. The data presented herein substantiated the results from the previous studies showing that pterostilbene improved the TNF-α-induced impairment of autophagic flux by upregulating the protein expression of LAMP2 and Rab7 and the number of autolysosomes in Caco-2 cells. In addition, chloroquine treatment abrogated the positive roles of pterostilbene in inflammatory responses and cell apoptosis in the TNF-α-treated cells. These findings suggest that autophagosome-lysosome fusion plays a pivotal role in the pterostilbene-mediated protection against colonic inflammation.

As the first physical and immunological protective barrier, intestinal epithelial cells separate the host from the external environment and prevent the invasion of bacteria, viruses, and endotoxins [52]. Defects in the intestinal barrier may therefore allow these undesired antigens to cross the intestinal epithelium and increase the risk of intestinal inflammation. Numerous studies have substantiated the potency of pterostilbene in protecting against intestinal barrier damage [22, 30, 53]. Here, pterostilbene treatment greatly alleviated the deficiencies in goblet cell quantity and the secretion of mucin 2 and TFF3 in the IUGR colon. It also upregulated the protein levels of occludin and ZO-1 and consequently led to a decrease in intestinal epithelial permeability in both the IUGR colon and the TNF-α-exposed Caco-2 cells. It is worthwhile to mention that ER stress inducer tunicamycin and the autophagic flux inhibitor chloroquine largely suppressed the pterostilbene-mediated beneficial effects on the intestinal epithelium permeability and intestinal tight junction protein. These findings indicate that the inhibition of ER stress and the promotion of autophagic flux are key mechanism by which pterostilbene prevents colon barrier dysfunction.

## Conclusion

This study highlights the roles of ER stress and impaired autophagic flux as part of the mechanism of the IUGR-induced colon inflammation. Importantly, pterostilbene effectively overcomes ER stress, restores autophagic flux, and further mitigates inflammatory responses and intestinal barrier dysfunction in both the IUGR colon and the TNF-α-treated Caco-2 cells. These findings may broaden the understanding of how pterostilbene protects against colon inflammation and help in development of novel nutrition strategies for IUGR animals to improve intestinal health.

## Supplementary Information

**Additional file 1: Table S1.** Composition and nutrient levels of the basal diet.

**Additional file 2: Table S2.** Primer sequences for quantitative real-time PCR and real-time PCR analyses.

## Data Availability

The data used to support the findings of the present study are available from the corresponding author upon request.

## Abbreviations

AdPlus-mCherry-GFP-LC3B: Adenovirus expressing mCherry-GFP-LC3B fusion protein recognizing CD46
ANOVA: Analysis of variance
ATF4: Activating transcription factor 4
ATF6: Activating transcription factor 6
BSA: Bovine serum albumin
BW: Birth weight
CHOP: CCAAT/enhancer binding protein homologous protein
c-Casp12: Cleaved caspase 12
DMEM: Dulbecco’s modified Eagle’s medium
eIF2α: Eukaryotic translational initiation factor 2 alpha
ELISA: Enzyme-linked immunosorbent assay
ER: Endoplasmic reticulum
FBS: Fetal bovine serum
GAPDH: Glyceraldehyde phosphate dehydrogenase
GRP78: Glucose-regulated protein 78
GRP94: Glucose-regulated protein 94
H&E: Hematoxylin and eosin
IRE1: Inositol-requiring kinase 1
IUGR: Intrauterine growth retardation
IL: Interleukin
LAMP2: Lysosomal associated membrane protein 2
LPS: Lipopolysaccharide
MPO: Myeloperoxidase
NBW: Normal birth weight
NF-κB p65: Nuclear factor kappa B p65
PERK: Protein kinase RNA-like ER kinase
qRT-PCR: Quantitative real-time PCR
sXBP-1: Spliced X-box binding protein-1
TER: Transepithelial electrical resistance
TFF3: Trefoil factor 3
TNF-α: Tumor necrosis factor alpha
TUNEL: Terminal deoxynucleotidyl transferase dUTP nick end labeling
UPR: Unfolded protein responses
ZO-1: Zonula occludens 1
3MA: 3-methyladenine
4PBA: 4-phenylbutyric acid
