# Supplementary material for: Pterostilbene attenuates intrauterine growth retardation-induced colon inflammation in piglets by modulating endoplasmic reticulum stress and autophagy
Source: J Anim Sci Biotechnol. 2022 Nov 4;13:125. doi: 10.1186/s40104-022-00780-6 (PMC9635184; doi:10.1186/s40104-022-00780-6)
Supplement: Supplementary file 1 — Additional file 1: Table S1. Composition and nutrient levels of the basal diet. [file 40104_2022_780_MOESM1_ESM.docx]

**Table S1** Composition and nutrient levels of the basal diet (%, as-fed basis unless otherwise stated)

| Items | Contents |
| --- | --- |
| Maize | 62.78 |
| Soybean meal | 15.00 |
| Fermented soybean meal | 7.00 |
| Extruded soybean | 7.00 |
| Soy protein isolate | 1.30 |
| Soyabean oil | 2.00 |
| CaHPO_4_ | 1.80 |
| Limestone | 0.80 |
| Salt | 0.35 |
| *L*-lysine-HCl (78%) | 0.52 |
| *L*-methionine | 0.13 |
| *L*-threonine | 0.15 |
| *L*-isoleucine | 0.10 |
| *L*-tryptophan | 0.01 |
| *L*-histidine | 0.01 |
| Calcium propionate (50%) | 0.05 |
| Premix^a^ | 1.00 |
| Total | 100.00 |
| Nutrient levels^b^ |  |
| Digestible energy, Mcal/kg | 3.47 |
| Metabolizable energy, Mcal/kg | 3.30 |
| Crude protein | 20.36 |
| Total lysine | 1.51 |
| Total methionine | 0.46 |
| Total methionine + cystine | 0.86 |
| Total threonine | 0.94 |
| Total tryptophan | 0.40 |
| Total histidine | 0.77 |
| Total isoleucine | 0.79 |
| Total valine | 1.20 |
| Total calcium | 0.82 |
| Total phosphorus | 0.65 |

^a^Provide the following per kg complete diet: Vitamin A, 8000 IU; Vitamin D_3_, 3000 IU; Vitamin E, 20 IU; Vitamin K_3_, 3 mg; Vitamin B_1_, 2 mg; Vitamin B_2_, 5 mg; Vitamin B_6_, 7 mg; Vitamin B_12_, 0.02 mg; Niacin, 30 mg; Pantothenic acid, 15 mg; Folic acid, 0.3 mg; Biotin, 0.08 mg; Choline chloride, 500 mg; Fe (from ferrous sulfate), 110 mg; Cu (from copper sulfate), 7 mg; Mn (from manganese sulfate), 5 mg; Zn (from zinc sulfate), 110 mg; I (from calcium iodate), 0.3 mg; Se (from sodium selenite), 0.3 mg.

^b^All nutrient levels were analyzed values, except digestible energy and metabolizable energy
