# Supplementary material for: Pterostilbene attenuates intrauterine growth retardation-induced colon inflammation in piglets by modulating endoplasmic reticulum stress and autophagy
Source: J Anim Sci Biotechnol. 2022 Nov 4;13:125. doi: 10.1186/s40104-022-00780-6 (PMC9635184; doi:10.1186/s40104-022-00780-6)
Supplement: Supplementary file 2 — Additional file 2: Table S2. Primer sequences for quantitative real-time PCR and real-time PCR analyses. [file 40104_2022_780_MOESM2_ESM.docx]

**Table S2** Primer sequences of target and reference genes

| Genes | GenBank Accession No. | Sequence (5’ → 3’) | Product length, bp |
| --- | --- | --- | --- |
| Swine |  |  |  |
| *GRP78* | XM_021068830.1 | F: GGTAAGTGGGGTTGGTGGAA | 112 |
|  |  | R: CACGGCCATTCTTGAACACC |  |
| *GRP94* | NM_214103.1 | F: CAACACTGCGGTCAGGGTAT | 103 |
|  |  | R: ACCTTTGCATCGGGGTCAAT |  |
| *CHOP* | NM_001144845.1 | F: GCTGGAAAGCAACGCATGAA | 149 |
|  |  | R: ACCATCCGGTCAATCAGAGC |  |
| *ATF4* | NM_001123078.1 | F: GTGGCCAAGCACTTCAAACC | 137 |
|  |  | R: ATCCAGTCTGTCCCGGAGAA |  |
| *sXBP1* | NM_001271738.1 | F: GGAGTTAAGACAGCGCTTGG | 142 |
|  |  | R: GAGATGTTCTGGAGGGGTGA |  |
| *GAPDH* | NM_001206359 | F: GTCGGTTGTGGATCTGACCT | 207 |
|  |  | R: TTGACGAAGTGGTCGTTGAG |  |
| Human |  |  |  |
| *TNF-α* | NM_000594.4 | F: AGAACTCACTGGGGCCTACA | 139 |
|  |  | R: AGGAAGGCCTAAGGTCCACT |  |
| *IL-1β* | XM_017003988.2 | F: AAGCCATAAAAACAGCGAGGG | 146 |
|  |  | R: GGGCCATCAGCTTCAAAGAAC |  |
| *IL-6* | NM_000600.5 | F: CCACCGGGAACGAAAGAGAA | 92 |
|  |  | R: GAGAAGGCAACTGGACCGAA |  |
| *XBP1* | NM_001079539.2 | F: TTGCTGAAGAGGAGGCGGAAG | 184/210 |
|  |  | R: GGTCCAAGTTGTCCAGAATGC |  |
| *GAPDH* | NM_001256799.3 | F: AATGGGCAGCCGTTAGGAAA | 168 |
|  |  | R: GCGCCCAATACGACCAAATC |  |

*ATF4*: activating transcription factor 4; *CHOP*: CCAAT/enhancer binding protein homologous protein; *GAPDH*: glyceraldehyde phosphate dehydrogenase; *GRP78*: glucose-regulated protein 78; *GRP94*: glucose-regulated protein 94; *IL-1β*: interleukin-1 beta; *IL-6*: interleukin-6; *sXBP-1*: spliced X-box binding protein-1; *TNF-α*: tumor necrosis factor alpha; *XBP-1*: X-box binding protein-1.
